# Multiple failed intubation attempts are associated with decreased success rates on the first rescue intubation in the emergency department: a retrospective analysis of multicentre observational data

**DOI:** 10.1186/s13049-014-0085-8

**Published:** 2015-01-16

**Authors:** Tadahiro Goto, Koichiro Gibo, Yusuke Hagiwara, Hiroshi Morita, David FM Brown, Calvin A Brown, Kohei Hasegawa, on behalf of the Japanese Emergency Medicine Network Investigators

**Affiliations:** Department of Emergency Medicine, University of Fukui Hospital, 23-3, Shimoaiduki, Matsuoka, Eiheiji, Yoshida, Fukui, 910-1193 Japan; Department of Emergency Medicine, Okinawa Prefectural Chubu Hospital, Miyazato, Uruma, Okinawa 904-2293 Japan; Division of Pediatric Emergency Medicine, Department of Pediatric Emergency and Critical Care Medicine, Tokyo Metropolitan Children’s Medical Centre, 2-8-29 Musashidai, Fuchu, Tokyo 183-8561 Japan; Department of Emergency Medicine, Massachusetts General Hospital, Harvard Medical School Boston, Boston, Massachusetts USA; Department of Emergency Medicine, Brigham and Women’s Hospital, Harvard Medical School, Boston, Massachusetts USA

**Keywords:** Intubation, Failed intubation, Rescue intubation, Success rate, Emergency department

## Abstract

**Background:**

Although the international guidelines emphasize early and systematic use of rescue intubation techniques, there is little evidence to support this notion. We aimed to test the hypothesis that preceding multiple failed intubation attempts are associated with a decreased success rate on the first rescue intubation in emergency departments (EDs).

**Methods:**

We analysed data from two multicentre prospective registries designed to characterize current ED airway management in Japan between April 2010 and June 2013. All patients who underwent a rescue intubation after a failed attempt or a series of failed attempts were included for the analysis. Multiple failed intubation attempts were defined as ≥2 consecutive failed intubation attempts before a rescue intubation. Primary outcome measure was success rate on the first rescue intubation attempt.

**Results:**

Of 6,273 consecutive patients, 1,151 underwent a rescue intubation. The success rate on the first rescue intubation attempt declined as the number of preceding failed intubation attempts increased (81% [95% CI, 79%-84%] after one failed attempt; 71% [95% CI, 66%-76%] after two failed attempts; 67% [95% CI, 55%-78%] after three or more failed attempts; P_trend_ <0.001). In the multivariable analysis adjusting for age, sex, principal indication, change in methods, devices, and intubator specialty, and clustering of patients within EDs, success rate on the first rescue intubation after two failed attempts was significantly lower (OR, 0.56; 95% CI, 0.41-0.77) compared to that after one failed attempt. Similarly, success rate on the first rescue intubation attempt after three or more failed attempts was significantly lower (OR, 0.49; 95% CI, 0.25-0.94) compared to that after one failed attempt.

**Conclusion:**

Preceding multiple failed intubation attempts was independently associated with a decreased success rate on the first rescue intubation in the ED.

## Background

Successful intubation is a critical and central intervention in the emergency department (ED). In particular, the concept of first pass success is frequently promoted as the goal of emergency airway management [1-4]. However, first intubation attempts in EDs are often unsuccessful; the literature documents that 17% to 29% of all ED intubations require two or more intubation attempts [4-8].

Given the emerging evidence that repetitive intubation attempts are associated with an increased risk of adverse events in the ED [3,4], early and successful rescue intubation approaches – e.g., use of alternative methods, devices, and intubators – become important. The international anaesthesia consensus statement emphasizes early use of rescue intubation approaches in the airway management [9-12], and several studies from the anaesthesia and pre-hospital literature support this approach [13-16]. However, to the best of our knowledge, there is no evidence to support or refute the early use of rescue intubation in the ED.

To address this knowledge gap in the literature, by using large prospective multicentre registry data of emergency airway management, we aimed to examine whether preceding multiple failed intubation attempts are associated with a decreased success rate on the first rescue intubation in the ED.

## Methods

### Study design and setting

We conducted a retrospective analysis of the Japanese Emergency Airway Network (JEAN) 1 and 2 registries. These multicentre prospective data registries were designed to describe current ED airway management across Japan. The study setting, methods of data collection, and measured variables of the JEAN 1 registry have been reported elsewhere [3,7,17,18]. Briefly, JEAN 1 was a consortium of 13 academic and community medical centres from different geographic regions across Japan. The participating institutions were certified as Level I (n = 11) or Level II equivalent (n = 2) trauma centres. These EDs had a median of 25,000 patient visits in the ED per year (range, 4,200 - 67,000). After the completion of JEAN 1 in March 2012, the JEAN 2 was initiated in April 2012.

JEAN 2 registry is also a prospective multicentre registry that aims to investigate the emergency airway management in the EDs. The study design and methods of data collection were similar to JEAN 1. JEAN 2 was a consortium of 11 academic and community medical centres across Japan. The participating institutions were certified as Level I (n = 9) or Level II equivalent (n = 2) trauma centres. These EDs had a median ED census of 30,000 patient visits per year (range, 14,000-67,000).

The EDs of all JEAN 1 and JEAN 2 participating centres were staffed by emergency medicine physicians, and all but one ED in JEAN 1 were affiliated with an emergency medicine residency training program. Non-emergency medicine residents also rotated through all EDs and participated in intubations. Paediatric patients were treated in all EDs. Each ED maintained individual protocols for the procedures and policies for emergency airway management. Airway management was performed by attending physicians, or by residents at the discretion of attending physicians in the EDs. The institutional ethics committee at each participating institution approved the study with waiver of informed consent.

### Participants

The registries collected information on all adult and paediatric patients who underwent intubation attempts in the ED between April 2010 and June 2013. Among these patients, those who underwent a rescue intubation after a failed attempt or a series of failed attempts were eligible for this analysis. We excluded patients who died before achieving intubation success, and those who were intubated by unknown intubator status or with using unknown methods or devices.

### Data collection and processing

After each intubation encounter in the ED, the intubator completed a standardized data collection form. The measured variables were age, sex, principal indication for intubation, methods of intubation, all medications used to facilitate intubation, intubator’s level of training and specialty, number of intubation attempts, intubation success or failure, and associated adverse events [3,7,17,18]. The compliance of data form completion was monitored by site investigators with reviewing professional billing records. Where the data were missing or contradicting, the involved physicians were interviewed by site investigators to ascertain airway management details. These post hoc interviews occurred within two weeks of the patient encounter.

### Outcome measurements

The outcome of interest was the success rate on the first rescue intubation. We defined a “first rescue intubation” as an intubation with a change in intubators, devices, and/or methods after a failed initial attempt or a series of failed attempts [5]. For example, when the intubator failed the initial intubation attempt with the use of sedative and attempted the second intubation with RSI, the following attempt with RSI was considered as “first rescue intubation.” In this study, we focused solely on first rescue intubation attempts given the clinical importance of early rescue success in EDs [4,19]. An oral attempt was defined as a single insertion of a laryngoscope (or other devices) past the teeth. For nasal intubations, an attempt was defined as a single insertion of a tracheal tube past the turbinates. An attempt was successful if it resulted in a tracheal tube being placed through the vocal cords, with confirmation by quantitative or colorimetric end-tidal carbon dioxide monitoring [6,7]. The alternative methods after a failed intubation attempt included rapid sequence intubation (RSI), intubation with sedatives, nasal intubation, and cricothyrotomy/tracheotomy. The adjunctive devices included video laryngoscopes, fiberoptic bronchoscopes, other types of direct laryngoscope, combination of a gum elastic bougie with direct laryngoscope or video laryngoscope, and supraglottic devices.

### Statistical analysis

Summary statistics were presented by means (with standard deviations [SDs]), medians (with interquartile ranges [IQRs]), and proportions (with 95% confidence intervals [CIs]) as appropriate.

For the purpose of this retrospective analysis, we classified the eligible patients into three groups based on the number of failed intubation attempts before the first rescue intubation: one failed attempt, two failed attempts, and three or more failed attempts. We defined multiple failed intubation attempts as two or more consecutive failed intubation attempts before the first rescue intubation attempt. To examine the association between the number of failed attempts before the first rescue intubation and the success rate on the first rescue intubation, we constructed a random effects model with binomial response and with one failed attempt as the reference. We adjusted for a set of patient-level confounders that was chosen based on biological plausibility and *a priori* knowledge [3,5,6,8,20], and account for the clustering of patients at the ED level. These patient-level variables included age, sex, principal indication for intubation, change in methods of intubation, change in devices, and change in intubator. We treated the number of failed intubation as a categorical variable after checking the linearity assumption. Similarly, age variables also treated as the categorical variable after checking the linearity assumption. Clinically meaningful interactions were tested as a group to avoid inflating type I error. Specifically, interactions between an indication for intubation and (1) a change in methods of intubation, (2) a change in devices of intubation, and (3) a change in intubator were tested using the likelihood ratio test. However, these interactions were not statistically significant, and therefore were not included in the final model (result not shown).

In sensitivity analysis, to assess the consistency of the association between the number of failed attempts and success rates on the first rescue intubation, we repeated the multivariable analysis, modelling with the number of failed attempts as dichotomous (2 or more vs. 1) and ordinal variables. In addition, to assess whether the success rate improves or declines with an increase in the number of failed attempts before the first rescue intubation, we used the Cochran-Armitage test. Summary statistics were conducted with JMP statistical software (version 10.0.2; SAS Institute, Inc., Cary, NC). The random effects model was conducted with R software version 3.0.3 (R Development Core Team, Vienna, Austria), with the lme4 package to fit the random effects models [21]. Two-sided P < 0.05 was considered statistically significant.

## Results

**Figure 1:**
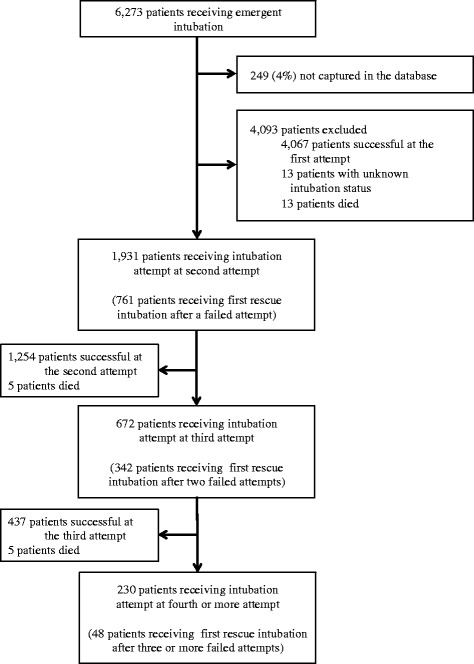
**Patients receiving intubations in emergency departments, according the number of failed intubation attempts.** All of the 23 patients who died during intubation had cardiac arrest as the primary indication of intubation (i.e., these patients had cardiac arrest prior to the intubation attempts).

During the study period, a total of 6,273 patients required emergency airway management in 15 EDs. Among these, the database recorded 6,024 patients (capture rate, 96%). We excluded 4,093 patients with successful first intubation attempts, with unknown airway management status, and those who died before achieving intubation success in the ED (Figure 1). Of the remaining 1,931 patients, 1,151 patients underwent the first rescue intubation after one or more failed intubation attempts, and were eligible for the current analysis.

Overall, the median age of the patients was 65 years and 37% were female. The primary indication for intubation was medical in 80%, and cardiac arrest in 34%. Patient characteristics differed across the patient groups according to the number of failed attempts (Table 1). For example, the patients with two or more (multiple) consecutive failed attempts had a higher proportion of medical indications for intubation compared to the patients who underwent a rescue intubation after one failed attempt.

**Table 1:** **Characteristics of 1151 patients receiving rescue intubation according the number of failed attempts**

**Patient characteristics at first rescue intubations**	**Number of failed attempts before first rescue intubation**		
	**1**	**2**	**3 or more**
	**(n = 761)**	**(n = 342)**	**(n = 48)**
Age, median (IQR), y	65 (49–77)	67 (54–78)	64 (43–74)
Age ≥ 18 years	736 (97)	330 (96)	44 (92)
Female sex	283 (37)	129 (38)	16 (33)
Primary indication*			
Medical encounters	585 (77)	290 (85)	44 (92)
Cardiac arrest	220 (29)	101 (30)	9 (19)
Altered mental status	186 (25)	82 (24)	18 (38)
Respiratory failure	102 (13)	69 (20)	12 (25)
Airway obstruction	26 (3)	8 (2)	4 (8)
Shock	49 (6)	29 (8)	1 (2)
Other medical	2 (1)	1 (1)	0 (0)
Trauma encounters	176 (23)	52 (15)	4 (8)
Traumatic arrest	57 (8)	8 (2)	0 (0)
Head trauma	50 (7)	18 (5)	2 (4)
Shock	21 (3)	8 (2)	1 (2)
Facial/neck trauma	18 (2)	9 (3)	0 (0)
Burn/inhalation	11 (1)	3 (1)	1 (2)
Other trauma	19 (3)	6 (2)	0 (0)

Abbreviation: IQR, interquartile range; SD, standard deviation.

Data were expressed as N (%) unless otherwise indicated.

*Percentages may not equal 100 due to rounding.

Overall, approximately half of the first rescue intubation attempts were performed without any medications, and rapid sequence intubation was used in an additional fifth of attempts. Direct laryngoscope was used in approximately 80% of rescue intubation attempts. Emergency physicians (including emergency medicine residents) performed approximately 80% of rescue intubation attempts. Similar to the patient characteristics, airway management at the first rescue intubation differed across the patient groups (Table 2). The patients with two or more consecutive failed attempts were more likely to receive the rescue intubation by an emergency medicine resident.

**Table 2:** **Airway management characteristics of 1151 patients receiving rescue intubation according the number of failed attempts**

**Management characteristics at first rescue intubations**	**Number of failed attempts before first rescue intubation**		
	**1**	**2**	**3 or more**
	**(n = 761)**	**(n = 342)**	**(n = 48)**
**Method**			
Oral without medication	394 (52)	162 (47)	22 (46)
Sedation without paralytics	136 (18)	85 (25)	15 (33)
Rapid sequence intubation	171 (22)	71 (21)	9 (19)
Surgical cricothyrotomy	17 (2)	8 (2)	1 (2)
Other*	43 (6)	16 (3)	1 (2)
**Device**			
Direct laryngoscope	601 (79)	276 (81)	39 (81)
Video laryngoscope	74 (10)	28 (8)	5 (10)
Other†	87 (11)	38 (11)	4 (8)
**Specialty of intubator**			
Transitional year resident‡	53 (7)	20 (6)	3 (6)
Emergency medicine resident	240 (32)	127 (37)	18 (38)
Emergency physician§	376 (49)	146 (43)	19 (40)
Other specialty	92 (12)	49 (14)	8 (17)
Major adverse events			
Cardiac arrest	3 (1)	1 (1)	1 (2)
Hypotension	9 (!)	8 (2)	1 (2)
Hypoxemia	11 (1)	2 (1)	1 (2)

Data were expressed as N (%) unless otherwise indicated.

*Defined as nasal intubation or paralytics without sedatives.

†Defined as flexible bronchoscope, a combination of a gum elastic bougie with direct laryngoscope or video laryngoscope, other types of direct laryngoscope, or supraglottic devices.

‡Defined as post graduate years 1 or 2.

§Defined as post graduate years ≥6.

**Figure 2:**
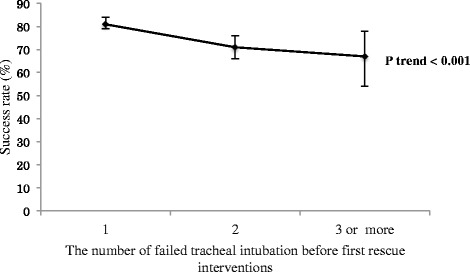
**First rescue intubation success rates, according to the number of failed intubation attempts.** I bars represent 95% confidence intervals. The success rate on first rescue intubation attempts declined as the number of failed intubation attempts increased (81% [95% CI, 79%-84%] after one failed attempt; 71% [95% CI, 66%-76%] after two failed attempts; 67% [95% CI, 55%-78%] after three or more failed attempts; P_trend_ <0.001).

The overall success rate on first rescue intubation attempts was 78% (95% CI, 75%- 80%). The success rate on first rescue intubation attempts declined as the number of preceding failed intubation attempts increased (81% [95% CI, 79%-84%] after one failed attempt; 71% [95% CI, 66%-76%] after two failed attempts; 67% [95% CI, 55%-78%] after three or more failed attempts; P_trend_ <0.001; Figure 2).

In multivariable analysis adjusting for age, sex, principal indication, change in methods, devices, and intubator specialty, and clustering of patients within EDs, the success rate on first rescue intubations after two failed attempts was significantly lower (OR, 0.56; 95% CI, 0.41-0.77; Table 3) compared to that after one failed attempt. Similarly, the success rate on first rescue intubations after three or more failed attempts were significantly lower (OR, 0.49; 95% CI, 0.25-0.94) compared to that after one failed attempt. In the sensitivity analysis, the adjusted association between multiple failed intubation attempts and decreased success rates on first rescue attempts persisted with the use of different definitions of multiple failed attempts (Table 3).

**Table 3:** **Multivariable associations of number of failed attempts before first rescue intubation with success rates**

	**Primary analysis**	**Sensitivity analysis**	
	**(No. of failed attempts as categorical variable)**	**(No. of failed attempts as dichotomous variable)**	**(No. of failed attempts as ordinal variable)**
**Variables**	OR (95% CI)	OR (95% CI)	OR (95% CI)
**Primary exposure**			
Number of failed attempts			
(categorical variable)			
1	1 [reference]	1 [reference]	1 [reference]
2	0.56 (0.41-0.77)	-	-
3 or more	0.49 (0.25-0.94)	-	-
≥2 (delayed intubation)*	-	0.55 (0.40-0.75)	-
Number of failed attempts (ordinal variable: OR per each incremental attempt)	-	-	0.60 (0.38-0.96)
**Age**			
0-19	0.84 (0.40-1.80)	0.84 (0.40-1.78)	0.84 (0.40-1.80)
20-39	0.82 (0.48-1.41)	0.82 (0.48-1.40)	0.82 (0.48-1.41)
40-59	1.09 (0.69-1.74)	1.09 (0.69-1.74)	1.09 (0.69-1.74)
60-79	1.22 (0.81-1.82)	1.22 (0.81-1.82)	1.22 (0.81-1.82)
80-99	1 [reference]	1 [reference]	1 [reference]
**Sex**			
Male	1 [reference]	1 [reference]	1 [reference]
Female	0.87 (0.64-1.18)	0.87 (0.64-1.18)	0.87 (0.64-1.18)
**Primary indication**			
Cardiac arrest	1 [reference]	1 [reference]	1 [reference]
Medical encounter	0.70 (0.49-1.01)	0.70 (0.49-1.00)	0.70 (0.49-1.01)
Trauma encounter	0.48 (0.30-0.76)	0.48 (0.30-0.76)	0.48 (0.30-0.76)
**Change in method**			
No change	1 [reference]	1 [reference]	1 [reference]
Rapid sequence intubation	0.60 (0.26-1.41)	0.61 (0.26-1.41)	0.61 (0.26-1.41)
Sedation without paralytics	0.26 (0.08-0.88)	0.26 (0.08-0.88)	0.26 (0.08-0.88)
Surgical cricothyrotomy	1.96 (0.42-9.07)	1.96 (0.42-9.04)	1.96 (0.42-9.04)
Others†	0.82 (0.23-2.95)	0.81 (0.22-2.94)	0.82 (0.23-2.95)
**Change in device**			
No change	1 [reference]	1 [reference]	1 [reference]
Direct laryngoscope	1.51 (0.59-3.86)	1.50 (0.59-3.85)	1.51 (0.59-3.86)
Gum elastic bougie	0.85 (0.41-1.77)	0.85 (0.41-1.77)	0.85 (0.41-1.77)
Video laryngoscope	0.47 (0.22-0.96)	0.46 (0.22-0.96)	0.47 (0.22-0.96)
Fiberoptic bronchoscope	0.23 (0.06-0.93)	0.23 (0.06-0.94)	0.23 (0.06-0.93)
Others‡	1.87 (0.20-17.39)	1.89 (0.20-17.59)	1.87 (0.20-17.45)
**Change in intubator**			
No change	1 [reference]	1 [reference]	1 [reference]
Transitional year resident§	0.25 (0.09-0.65)	0.25 (0.09-0.65)	0.25 (0.09-0.65)
Emergency medicine resident	0.89 (0.45-1.77)	0.89 (0.45-1.76)	0.90 (0.45-1.78)
Emergency physician¶	1.15 (0.60-2.19)	1.14 (0.60-2.18)	1.15 (0.60-2.19)
Other specialty	0.70 (0.34-1.47)	0.70 (0.34-1.47)	0.70 (0.34-1.47)

Abbreviations: OR, odds ratio; CI, confidence intervals.

*Delayed rescue intubation was defined as the first rescue intubation attempt after two or more failed attempts.

†Defined as nasal intubation or paralytics without sedatives.

‡Defined as other types of direct laryngoscope, or supraglottic devices.

§Defined as post graduate years 1 or 2.

¶Defined as post graduate years ≥6.

## Discussion

Using the data from two large prospective multicentre observational registries of patients undergoing airway management in the EDs, we demonstrated that the success rate on the first rescue intubation attempt declined as the number of preceding failed intubation attempts increased. To the best of our knowledge, this is the first demonstration of an association between multiple failed intubation attempts and success rates at the rescue intubations in ED airway management.

Airway management has been advocated as the first step for critically ill patients in the ED. However, most of scientific knowledge of airway management originates from out-of-hospital or anaesthesia settings [10,22-28]. Generalizability of these data to the ED setting might be limited because of the difference in patient population (e.g., most of ED patients undergoing airway management are critically ill and have a limited physiologic reserve), setting (i.e., EDs are less controlled setting), individual training level of intubators, and resources. Many vital questions about ED-based airway management to avoid ineffective or potentially harmful interventions remains to be elucidated [29]. Although high-quality prospective clinical trials will provide more rigorous evidence to advance the science of ED airway management, it is often difficult to study airway management in critically ill patients in the ED setting. In the last decade, large prospective registries represented by the National Emergency Airway Registry, Korean Emergency Airway Management Registry, and JEAN registries have provided scientific evidence of emergency airway management [5-7,20]. These registries allow insights into not only individual and group performance but also infrequent outcomes such as failed intubations and rescue intubations. Although rescue intubation is deemed to be important [3,30-32], previous studies in the ED setting only examined the characteristics of rescue intubations and the predictors for successful rescue attempts after one failed attempt (i.e., not after multiple failed intubation attempts) [8,19].

The American Society of Anesthesiologists guidelines recommend the early use of alternative approaches after multiple failed intubations [10]. Several studies in the non-ED setting support this approach [13-15,33-35]. For example, two small single-centre studies reported successful implementations of the predefined algorisms that use alternative devices (e.g., gum elastic bougie and supraglottic airways) after two failed laryngoscopic attempts, both in the operating room (n = 100) [34] and prehospital settings (n = 160) [35]. Another single-centre observational study reported that the use of video laryngoscope was effective as a primary rescue device in failed intubation attempts in the ED setting [36]. These studies collectively suggest the importance of early and systematic approaches in rescue intubations after failed intubation attempts. Our data from the large multicentre registries extend these prior investigations by demonstrating the independent association between the number of preceding failed intubation attempts and lower success rates on the first rescue intubation. Our inference supports the recommendation to minimizing the number of initial intubation attempts and focus on changing or optimizing the next attempt to maximize chances of success.

If not directly causative, multiple failed attempts before the rescue attempt have a plausible relationship with the observed lower success rates on the first rescue intubation. Potential reasons for the observed association include the difference in patient characteristics, rescue methods, devices, and providers among the groups according the number of failed attempts. However, the observed association persisted after conditioning on these factors. Alternatively, multiple intubation attempts are known to result in direct airway trauma [3,4], thereby interfering with airway visualization. Additionally, multiple failed attempts may be an identifiable surrogate marker for a number of factors – e.g., unanticipated difficult airway, limited provider awareness of difficult airways, lack of resource or education for emergency airway management – that affect success rates but are difficult to quantify individually.

Our findings have several implications for airway management in the ED. For clinicians, our data support the strategy limiting the number of intubation attempts and the conventional wisdom emphasizing the early and systematic use of rescue interventions during ED intubation efforts. However, the use of patient characteristics to assess difficult airways and the evidence to guide the provision of optimal airway management in the ED remain limited. For emergency researchers, our findings should underscore the importance of high-quality research into risk stratification and integrated rescue intubation approach, coupled with dissemination of these findings to improve care for patients in the ED.

Our data consist predominantly of academic and urban EDs in Japan where emergency airway management is highly variable across the EDs [7]. Thus, similar studies with data from other countries with different training systems may result in different findings. Although one may surmise a limited generalizability of our inferences, the observed association between the number of preceding failed intubation attempts and decreased rescue success rates was large and persisted across various statistical assumptions. In addition, there are plausible mechanisms to support this conclusion. While we acknowledge that validations of our study findings in systems with established emergency medicine education and training are warranted, we believe that our inferences are clinically plausible and likely applicable to different practice settings.

In sum, based on data from two prospective multicentre observational studies of patients undergoing airway management in the ED, we demonstrated that an increased number of failed attempts was independently associated with a decreased success rate of the first rescue intubation attempt. Our results support the early and systematic use of rescue intubation techniques for emergency airway management, including the use of an experience intubator [5], RSI [37], and video laryngoscope [36,38] supported by the literature.

## Limitations

The current study has several limitations. There are inherent limitations related to the use of surveillance data. The surveillance system used in these registries is subject to self-reporting bias, thereby resulting in an overestimation of intubation success rates. Although independent real-time monitoring of airway management is difficult to perform in the ED, we used the previously applied self-reporting systems with standardized data forms and high capture rates [6,7]. Therefore, we believe that our data represent the best available data.

Second, the JEAN registries were not designed to measure intubation-associated adverse events according to each intubation attempt; therefore, we were unable to examine associations between delayed rescue intubations and adverse events.

Third, as with any observational study, the observed association between the multiple failed intubation attempts and lower rescue success rates does not necessarily prove causality and might be confounded by unmeasured factors. Potential confounding factors include underlying comorbidities, difficulty in intubation (e.g., anatomical deformities and obesity), individual intubator training levels and skill sets, and institution-level characteristics. However, the adjustment for the specialty as a proxy of individual training levels might have partially addressed this potential confounding.

Fourth, a smaller proportion of patients in our registries underwent intubation attempts using RSI and video laryngoscope compared to the previous studies in different practice settings [4-6,8,16,20], owing to, at least in part, a high degree of variation in airway management practices [7]. To address this concern, we fitted mixed model accounting for the potential clustering of patients at the ED level. Interestingly, we found that neither the use of RSI nor video laryngoscope had a higher odds of first rescue success, both of which were not consistent with the literature [8,28,36]. The potential explanation of these findings includes the presence of residual confounders between these factors and rescue success rates, the limited statistical power owing to the small number of RSI and video laryngoscope use, the use of video laryngoscope by novices, and random errors.

Fifth, because our sample consisted predominantly of EDs affiliated with emergency medicine residency programs in Japan, our inferences may not be generalizable to the other healthcare settings (e.g., emergency airway management performed by experienced anaesthesiologists, with a higher use of RSI and video laryngoscope). However, the observed association has plausible mechanisms and persisted across different analytic assumptions. Although formal validation of the study in other healthcare settings is warranted, our inference is likely present in different practice settings.

Finally, our objective was not to examine predictors of the success rates by multiple rescue attempts but those on first rescue attempts. However, as the literature documented that intubation-associated adverse event rates accelerate with the increased number of intubation attempts [3,4], our observations are of direct relevance to the development of more effective emergency airway management for critically ill patients.

## Abbreviations

ED: Emergency department
JEAN: Japanese emergency airway network
RSI: Rapid sequence intubation

## References

[1] Mort TC (2004). Emergency tracheal intubation: complications associated with repeated laryngoscopic attempts. Anesth Analg.

[2] Levitan R (2005). The importance of a laryngoscopy strategy and optimal conditions in emergency intubation. Anesth Analg.

[3] Hasegawa K, Shigemitsu K, Hagiwara Y, Chiba T, Watase H, Brown CA (2012). Association between repeated intubation attempts and adverse events in emergency departments: an analysis of a multicenter prospective observational study. Ann Emerg Med.

[4] Sakles JC, Chiu S, Mosier J, Walker C, Stolz U (2013). The importance of first pass success when performing orotracheal intubation in the emergency department. Acad Emerg Med.

[5] Sagarin MJ, Barton ED, Chng YM, Walls RM, National Emergency Airway Registry I (2005). Airway management by US and Canadian emergency medicine residents: a multicenter analysis of more than 6,000 endotracheal intubation attempts. Ann Emerg Med.

[6] Walls RM, Brown CA, Bair AE, Pallin DJ, Investigators NI (2011). Emergency airway management: a multi-center report of 8937 emergency department intubations. J Emerg Med.

[7] Hasegawa K, Hagiwara Y, Chiba T, Watase H, Walls RM, Brown DF (2012). Emergency airway management in Japan: Interim analysis of a multi-center prospective observational study. Resuscitation.

[8] Kim JH, Kim YM, Choi HJ, Je SM, Kim E, Korean Emergency Airway Management Registry I (2013). Factors associated with successful second and third intubation attempts in the ED. Am J Emerg Med.

[9] Practice guidelines for management of the difficult airway (1993). A report by the American Society of Anesthesiologists Task Force on Management of the Difficult Airway. Anesthesiology.

[10] Apfelbaum JL, Hagberg CA, Caplan RA, Blitt CD, Connis RT, Nickinovich DG (2013). Practice guidelines for management of the difficult airway: an updated report by the American Society of Anesthesiologists Task Force on Management of the Difficult Airway. Anesthesiology.

[11] Law JA, Broemling N, Cooper RM, Drolet P, Duggan LV, Griesdale DE (2013). The difficult airway with recommendations for management–part 1–difficult tracheal intubation encountered in an unconscious/induced patient. Can J Anaesth.

[12] Walls RM, Murphy M (2012). Manual of Emergency Airway Management.

[13] Dimitriou V, Voyagis GS, Brimacombe JR (2002). Flexible lightwand-guided tracheal intubation with the intubating laryngeal mask Fastrach in adults after unpredicted failed laryngoscope-guided tracheal intubation. Anesthesiology.

[14] Heidegger T, Gerig HJ, Ulrich B, Kreienbuhl G (2001). Validation of a simple algorithm for tracheal intubation: daily practice is the key to success in emergencies–an analysis of 13,248 intubations. Anesth Analg.

[15] Parmet JL, Colonna-Romano P, Horrow JC, Miller F, Gonzales J, Rosenberg H (1998). The laryngeal mask airway reliably provides rescue ventilation in cases of unanticipated difficult tracheal intubation along with difficult mask ventilation. Anesth Analg.

[16] Levitan RM, Heitz JW, Sweeney M, Cooper RM (2011). The complexities of tracheal intubation with direct laryngoscopy and alternative intubation devices. Ann Emerg Med.

[17] Hasegawa K, Hagiwara Y, Imamura T, Chiba T, Watase H, Brown 3rd CA, et al. Increased incidence of hypotension in elderly patients who underwent emergency airway management: an analysis of a multi-centre prospective observational study. Int J Emerg Med. 2013;6:12. 10.1186/1865-1380-6-12 PMC3640904 23618100

[18] Imamura T, Brown CA, Ofuchi H, Yamagami H, Branch J, Hagiwara Y (2013). Emergency airway management in geriatric and younger patients: analysis of a multicenter prospective observational study. Am J Emerg Med.

[19] Bair AE, Filbin MR, Kulkarni RG, Walls RM (2002). The failed intubation attempt in the emergency department: analysis of prevalence, rescue techniques, and personnel. J Emerg Med.

[20] Kim C, Kang HG, Lim TH, Choi BY, Shin YJ, Choi HJ (2013). What factors affect the success rate of the first attempt at endotracheal intubation in emergency departments?. Emerg Med J.

[21] **lme4: Linear mixed-effects models using Eigen and S4. R package version 1.0-5.** [http://CRAN.R-project.org/package=lme4]

[22] Dunford JV, Davis DP, Ochs M, Doney M, Hoyt DB (2003). Incidence of transient hypoxia and pulse rate reactivity during paramedic rapid sequence intubation. Ann Emerg Med.

[23] Wang HE, Peitzman AB, Cassidy LD, Adelson PD, Yealy DM (2004). Out-of-hospital endotracheal intubation and outcome after traumatic brain injury. Ann Emerg Med.

[24] Wang HE, Simeone SJ, Weaver MD, Callaway CW (2009). Interruptions in cardiopulmonary resuscitation from paramedic endotracheal intubation. Ann Emerg Med.

[25] Wang HE, Balasubramani GK, Cook LJ, Lave JR, Yealy DM (2010). Out-of-hospital endotracheal intubation experience and patient outcomes. Ann Emerg Med.

[26] Wang HE, Mann NC, Mears G, Jacobson K, Yealy DM (2011). Out-of-hospital airway management in the United States. Resuscitation.

[27] Hasegawa K, Hiraide A, Chang Y, Brown DF (2013). Association of prehospital advanced airway management with neurologic outcome and survival in patients with out-of-hospital cardiac arrest. JAMA.

[28] Lossius HM, Roislien J, Lockey DJ. Patient safety in pre-hospital emergency tracheal intubation: a comprehensive meta-analysis of the intubation success rates of EMS providers. Crit Care. 2012;16:R24. 10.1186/cc11189 PMC3396268 22325973

[29] Wang HE, Yealy DM (2014). Emergency airway research: using all tools to bridge the knowledge gaps. Ann Emerg Med.

[30] Stefanutto TB, Feiner J, Krombach J, Brown R, Caldwell JE (2012). Hemoglobin desaturation after propofol/remifentanil-induced apnea: a study of the recovery of spontaneous ventilation in healthy volunteers. Anesth Analg.

[31] Kim J, Kim K, Kim T, Rhee JE, Jo YH, Lee JH (2014). The clinical significance of a failed initial intubation attempt during emergency department resuscitation of out-of-hospital cardiac arrest patients. Resuscitation.

[32] Quinn AC, Milne D, Columb M, Gorton H, Knight M (2013). Failed tracheal intubation in obstetric anaesthesia: 2 yr national case–control study in the UK. Br J Anaesth.

[33] Crosby ET, Cooper RM, Douglas MJ, Doyle DJ, Hung OR, Labrecque P (1998). The unanticipated difficult airway with recommendations for management. Can J Anaesth.

[34] Combes X, Le Roux B, Suen P, Dumerat M, Motamed C, Sauvat S (2004). Unanticipated difficult airway in anesthetized patients: prospective validation of a management algorithm. Anesthesiology.

[35] Combes X, Jabre P, Margenet A, Merle JC, Leroux B, Dru M (2011). Unanticipated difficult airway management in the prehospital emergency setting: prospective validation of an algorithm. Anesthesiology.

[36] Sakles JC, Mosier JM, Patanwala AE, Dicken JM, Kalin L, Javedani PP. The C-MAC(R) Video Laryngoscope Is Superior to the Direct Laryngoscope for the Rescue of Failed First-Attempt Intubations in the Emergency Department. J Emerg Med. 2014. [Epub ahead of print]. 10.1016/j.jemermed.2014.10.007 25498851

[37] Reynolds SF, Heffner J (2005). Airway management of the critically ill patient: rapid-sequence intubation. Chest.

[38] Sakles JC, Mosier J, Chiu S, Cosentino M, Kalin L (2012). A comparison of the C-MAC video laryngoscope to the Macintosh direct laryngoscope for intubation in the emergency department. Ann Emerg Med.

